# Intelligibility of audiovisual sentences drives multivoxel response patterns in human superior temporal cortex

**DOI:** 10.1016/j.neuroimage.2021.118796

**Published:** 2021-12-11

**Authors:** Johannes Rennig, Michael S Beauchamp

**Affiliations:** a Division of Neuropsychology, Center of Neurology, Hertie-Institute for Clinical Brain Research, University of Tübingen, Tübingen, Germany; b Department of Neurosurgery, Perelman School of Medicine, University of Pennsylvania, Richards Medical Research Building, A607, 3700 Hamilton Walk, Philadelphia, PA 19104-6016, United States

## Abstract

Regions of the human posterior superior temporal gyrus and sulcus (pSTG/S) respond to the visual mouth movements that constitute visual speech and the auditory vocalizations that constitute auditory speech, and neural responses in pSTG/S may underlie the perceptual benefit of visual speech for the comprehension of noisy auditory speech. We examined this possibility through the lens of multivoxel pattern responses in pSTG/S. BOLD fMRI data was collected from 22 participants presented with speech consisting of English sentences presented in five different formats: visual-only; auditory with and without added auditory noise; and audiovisual with and without auditory noise. Participants reported the intelligibility of each sentence with a button press and trials were sorted *post-hoc* into those that were more or less intelligible. Response patterns were measured in regions of the pSTG/S identified with an independent localizer. Noisy audiovisual sentences with very similar physical properties evoked very different response patterns depending on their intelligibility. When a noisy audiovisual sentence was reported as intelligible, the pattern was nearly identical to that elicited by clear audiovisual sentences. In contrast, an unintelligible noisy audiovisual sentence evoked a pattern like that of visual-only sentences. This effect was less pronounced for noisy auditory-only sentences, which evoked similar response patterns regardless of intelligibility. The successful integration of visual and auditory speech produces a characteristic neural signature in pSTG/S, highlighting the importance of this region in generating the perceptual benefit of visual speech.

## 1. Introduction

Enabling social interactions, including speech production and perception, is a key function of the human brain. Understanding speech is a complex computational problem that the brain solves using both visual information from the talker’s facial movements and auditory information from the talker’s voice. Visual speech information is particularly important under noisy listening conditions when auditory speech is difficult or impossible to understand alone (reviewed in Peelle and Sommers 2015).

The perceptual and neural mechanisms underlying the integration of auditory and visual speech are a subject of active investigation (Bernstein and Liebenthal, 2014; Hickok and Poeppel, 2007; O’Sullivan et al., 2021; Plass et al., 2020). In non-human primates, recordings from single neurons in pSTG/S respond to both auditory and visual social communication signals (Barraclough et al., 2005; Bruce et al., 1981; Dahl et al., 2009). In humans, small populations of neurons in pSTG/S recorded with intracranial electrodes respond to both auditory and visual speech (Karas et al., 2019; Rhone et al., 2016).

While the idea that pSTG/S integrates visual speech information with noisy auditory speech in the service of comprehension seems reasonable, it is supported by limited empirical evidence. A patient with a lesion of left pSTG/S had preserved audiovisual speech perception, although this could have been due to compensation by the right pSTG/S (Baum et al., 2012). The amplitude of responses in pSTG/S and the connectivity of pSTG/S are diminished in patients with autism spectrum disorder, possibly contributing to their language difficulties (Borowiak et al., 2020, 2018). In a recent study of healthy adults, repetitive transcranial magnetic stimulation (rTMS) was used to disrupt processing during perception of noisy auditory sentences (Kennedy-Higgins et al., 2020). rTMS of pSTG/S resulted in small but significant decreases (between one and two dB) in the ability to understand noisy speech.

However, a BOLD fMRI study of healthy adults failed to find evidence that pSTG/S integrates noisy auditory and visual speech. Bishop and Miller presented noisy audiovisual syllables and used *post hoc* sorting to separate trials into those that were understood and those that were not (Bishop and Miller, 2009). Differential responses to the two types of trials was observed in seventeen different brain areas, but pSTG/S was not of them.

The Bishop and Miller study suffers from two limitations. First, as in many published neuroimaging studies, Bishop and Miller used a volumetric group analysis in which each participant was aligned to a template brain and analysis was conducted at the group level. While pSTG/S was classified as a single cytoarchitectonic area by Brodmann (BA 22), high-resolution fMRI revealed small compartments within pSTG/S that selectively respond to auditory, visual and auditory-visual stimuli (Beauchamp et al., 2004a). The patchy organization of these compartments is idiosyncratic, meaning that given co-ordinate in standard space is likely to correspond to different compartments in different subjects. Volumetric group analysis ignores this variability and assumes that a given coordinate in standard space is functionally equivalent across participants, an assumption that can lead to incorrect inferences (Jiang et al., 2015).

Second, Bishop and Miller performed a univariate analysis, examining the response amplitude in *individual* voxels and regions of interest. However, in many circumstances, multivariate analyses of the pattern of activity across *multiple* voxels reveals information hidden from univariate analyses (Norman et al., 2006). Multivoxel analysis of responses to auditory-only speech has been used to show selectivity for specific speech features and talkers in auditory cortex (De Martino et al., 2008; Formisano et al., 2008) and sensitivity to speech intelligibility in pSTG/S (Okada et al., 2010).

Prompted by the limitations of the Bishop and Miller study, we set out to re-examine the relationship between comprehension of noisy audiovisual speech and BOLD responses in pSTG/S using an alternative approach. Instead of a volumetric group analysis, we used an individual subject analysis based on functional localizers. As with other pSTG/S localizers (Bernstein et al., 2011; Borowiak et al., 2018; Pelphrey et al., 2005), our pSTG/S localizer measured responses to silent visual stimuli, specifically videos of actors making mouth movements with silent videos of actors making eye movements (Zhu and Beauchamp, 2017). Even though the localizer contains only unisensory visual stimuli, it identifies regions of pSTG/S that respond to voices and prefer voices to environmental sounds during both fixation tasks and free-viewing of faces (Belin et al., 2000; Rennig and Beauchamp, 2018). Instead of univariate analysis, we applied multivoxel pattern analysis (Cox and Savoy, 2003; Norman et al., 2006) to examine the multivariate pattern of responses evoked by intelligible and unintelligible auditory and audiovisual speech in the pSTG/S.

## 2. Methods

Twenty-two healthy right-handed participants (14 females, mean age 25, range 18–34) with normal or corrected to normal vision and normal hearing provided written informed consent under an experimental protocol approved by the Committee for the Protection of Human Subjects of the Baylor College of Medicine, Houston, TX.

Participants were scanned in a 3 tesla Siemens Trio MRI scanner equipped with a 32-channel head coil at Baylor College of Medicine’s Core for Advanced MRI. Visual stimuli were presented on an MR compatible screen (BOLDscreen32, Cambridge Research Systems, Rochester, UK) placed behind the bore of the MR scanner and viewed through a mirror. Auditory stimuli were presented using high-fidelity MR compatible headphones (Sensimetrics, Malden, MA, USA). Behavioral responses were collected using a fiber-optic button response pad (Current Designs, Haverford, PA, USA) and eye movements were recorded during scanning using the Eye Link 1000 (SR Research Ltd., Ottawa, Ontario, Canada) with a sampling rate of 500 Hz. Stimuli were presented and synchronized with the MR data acquisition using Matlab (The Mathworks, Inc., Natick, MA, USA) with the Psychophysics Toolbox extensions (Brainard, 1997; Pelli, 1997).

### 2.1. Audiovisual speech stimuli

**Fig. 1.**
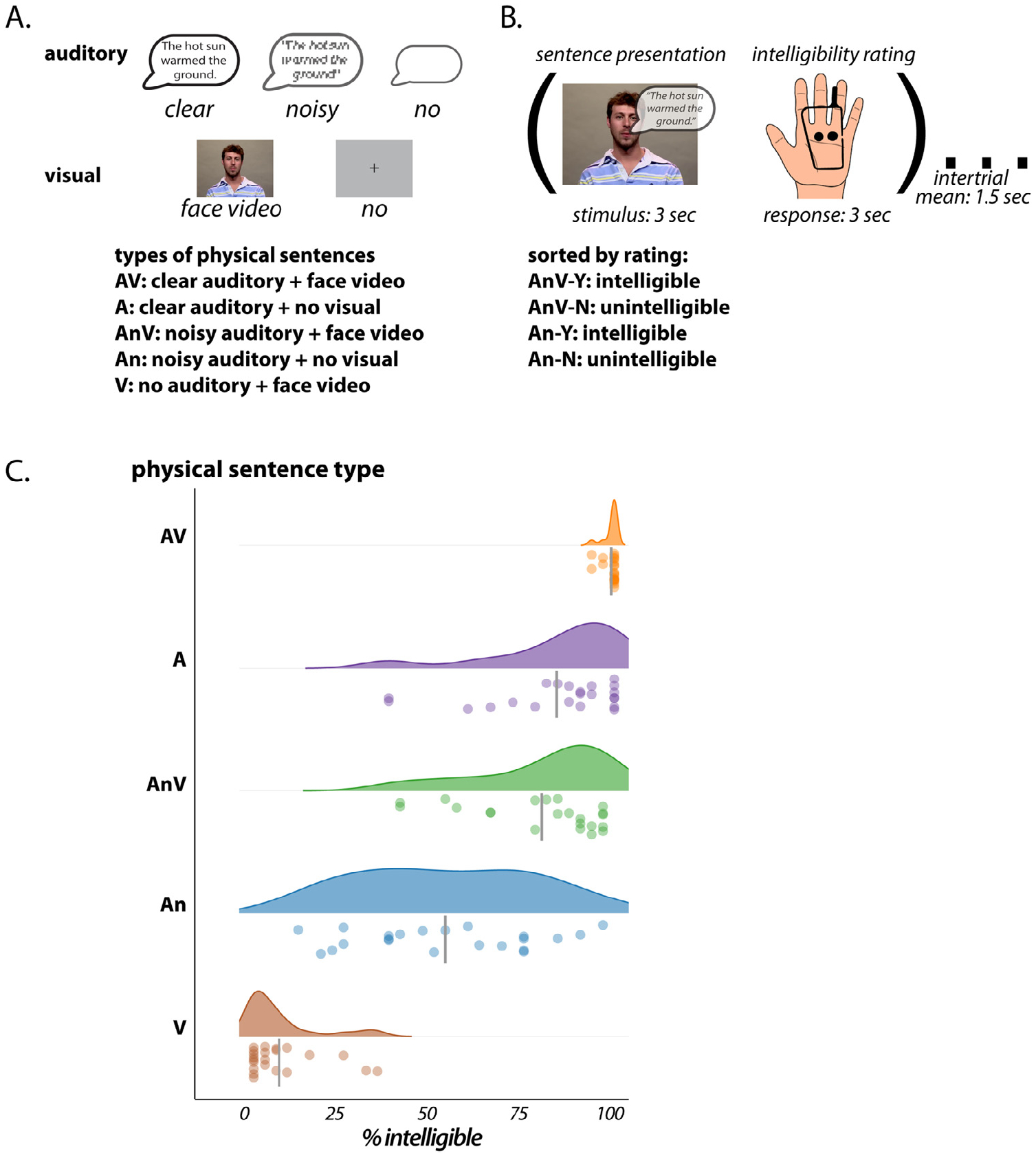
**A**. The auditory component of the stimulus consisted of a recording of a sentence without added noise (clear); with added pink noise (noisy); or silence (no). The visual component of the stimulus consisted of a video of the face of the talker speaking the sentence (face video) or a blank screen with fixation crosshairs (no). There were five types of physically different sentences consisting of different combinations of auditory and visual. **B**. Following the presentation of a sentence, participants rated the intelligibility of the sentence with a button press. Following a variable intertrial interval, the next trial began. Sentences containing a noisy auditory component (AnV, An) were *post hoc* sorted by intelligibility rating. **C.** For each physical sentence type, the percent of sentences rated as intelligible is shown with a raincloud plot (Allen et al., 2021). The top plot for each sentence type shows the probability density function, the bottom plot shows one symbol per participant (the percept of sentences of that type rated as intelligible by that participant). The vertical gray bar shows the mean across participants.

Audiovisual sentences (*e.g*. “the hot sun warmed the ground”) were recorded from a single male talker; the same stimulus set has been used in previous behavioral studies that used the same stimulus set (Rennig et al., 2020; Van Engen et al., 2017). The sentences were presented in five different formats (Fig. 1A): audiovisual (AV, video and clear audio), auditory (A, only clear audio), visual (V, only video), noisy audiovisual (AnV, video and noisy audio) and noisy auditory versions (An, only noisy audio). The original clear auditory recordings were normalized to equate root-mean-square amplitude across sentences, but no compression or normalization was done within each sentence.

To create noisy sentences, the original audio recordings were combined with pink noise. Pink noise is commonly used in studies of auditory function because it contains decreasing energy at increasing frequency, making it less aversive than white noise. Pink noise and the sentence audio track were normalized by the absolute value of the respective maximum, audio track_normalized_ = audio track_native_/max(abs(audio track_native_)). The power of the signal in the sentence audio track and the pink noise were determined and the signal-to-noise ratio (SNR) calculated as log*10*(power_signal_/power_noise_). The volume of the pink noise was increased or decreased iteratively to reach an SNR of −16 dB. The sentence audio track and pink noise were then summed and then renormalized to equalize the volume across all auditory sentences. In addition to the pink noise used to create the noisy auditory sentences, it should be noted that the echo-planar pulse sequence used for MR acquisition produced a constant background of moderate auditory noise in all conditions. The visual angle subtended by the face videos in the MR scanner was approximately 20° and the sound pressure level was approximately 80 dB.

### 2.2. Trial design

Each 6 s trial consisted of the presentation of a 3 s sentence (in one of 5 formats) followed by a 3 s response period during which participants made a button press to record their intelligibility judgment (Fig. 1B). Therefore, there was always a minimum of a 3 s interstimulus interval between sentences, corresponding to the duration of the response period. The order of the trials and the intertrial interval were set to a pseudo-random optimal sequence generated by the program *optseq2* (Dale et al., 1999, https://surfer.nmr.mgh.harvard.edu/optseq).

For the main fMRI experiment, four scan series were collected for each participant. Each series had total duration of 300 s. A rapid event-related design was used to present 40 sentence trials, with 8 sentences from each of the 5 different formats. 60 s of additional fixation baseline, distributed between the trials by the optimal sequencer to generate additional power for the “all stimulus *vs*. fixation baseline” contrast. This resulted in a mean intertrial interval of 1.5 s and a mean interstimulus interval of 4.5 s (3 s response window + intertrial interval).

### 2.3. Perceptual task and trial sorting

In the manuscript, we define “intelligible” using the colloquial sense of “comprehensible” or “able to be understood”. After each sentence was presented, participants rated intelligibility with a button press. The rating choices were: “understood everything” (all words in the sentence understood); “understood something” (at least one word in the sentence); “understood nothing” (no words in the sentence).

To minimize perceptual learning, sentences were never repeated within participants. For instance, presenting the same sentence in a clear format, followed later by presentation of the same sentence in a noisy format, would be expected to increase intelligibility of the primed noisy sentence, compared with a design in which the noisy sentence was not primed.

There were very few “understood everything” responses (8% in the An condition) so “understood everything” and “understood something” responses were grouped for analysis, resulting in two types of perceptually-sorted trials: “yes—some or all of the sentence intelligible” (Y) and “not at all intelligible” (N). This resulted in similar numbers of sentences in the two categories, critical for comparisons of the evoked brain responses. On a small fraction (4%) of trials, the participant did not respond. These trials were not analyzed.

### 2.4. Localizer *fMRI* experiment

The superior temporal cortex contains multiple, functionally heterogeneous regions, only some of which likely contribute to multisensory speech perception (Beauchamp, 2019; Beauchamp et al., 2004a). While large regions of pSTG/S respond to moving faces and voices, regions important for speech perception should respond more strongly to mouth movements. Regions of pSTG/S that prefer visually presented mouth movements respond strongly to auditory speech and prefer vocal sounds to non-vocal sounds (Zhu and Beauchamp, 2017). The stimuli of Zhu and Beauchamp (2017) were used for an independent localizer fMRI experiment. The localizer experiment used a different stimulus set than used in the main experiment (silent videos of actors making facial movements *vs*. audiovisual recordings of sentences). Each trial consisted of a silent 2 s video that showed the face of one of two actors making either a single mouth movement or a single eye movement. The video was followed by a 1 s response window, for a total trial duration of 3 s. Participants pressed a button to identify the actor in the video (two-alternative forced choice). This task was orthogonal to the presence of mouth or eye movements and was the same for all trials. Trials were organized into blocks of ten trials, either all “mouth” trials or all “eye” trials, for a total block length of 30 s. Each block was followed by 10 s of fixation baseline. Three mouth and three eye blocks were presented alternately during each scan series, for a total duration of 240 s. Two localizer scan series were collected for each participant, and the localizer scans always followed the main experiment.

### 2.5. MRI acquisition

Six echo-planar-imaging (EPI) scan series (four for the main experiment and two for the localizer) followed by two T1-weighted MP-RAGE anatomical volumes were collected from each participant. EPI data was acquired using a multi-slice echo planar imaging sequence (Setsompop et al., 2012): TR = 1500 ms, TE = 30 ms, flip angle = 72°, in-plane resolution of 2 × 2, 69 2 mm axial slices, multiband factor: 3, GRAPPA factor: 2.

### 2.6. Anatomical MRI analysis

The second MP-RAGE volume was aligned to the first MP-RAGE volume by a 6-parameter affine transformation with a mutual information cost function using the AFNI program *3dAllineate*. The aligned volumes were averaged to improve gray-white contrast and FreeSurfer was used to construct a cortical surface model (Dale et al., 1999a) which was visualized with the AFNI program *SUMA* (Argall et al., 2006).

### 2.7. *fMRI* analysis

All of the Siemens-format .IMA files from each scan series were concatenated into a single NiFTI file using the AFNI program *to3d* (Cox, 1996). Then, *afni_proc.py* was used for the remainder of the analysis. Briefly, all slices from each EPI brain volume were aligned in time to account for the timing of acquisition using the AFNI program *3dT-shift*. Then, co-registration (motion correction) was carried out using *align_epi_anat.py*. All EPI brain volumes were spatially aligned using the AFNI program *3dvolreg*. The EPI brain volumes were skull-stripped using the AFNI program *3dAutomask* and the average MP-RAGE brain volume was skull-stripped using the AFNI program *3dSkullStrip*. The skull-stripped EPI and MP-RAGE volumes were aligned using the AFNI program *3dAllineate* with a localized Pearson correlation cost function (Saad et al., 2009). The two co-registration transformations (within EPI, and between EPI and MP-RAGE) were concatenated and applied together, followed by blurring with a 3-dimensional Gaussian filter with a full-width at half-maximum of 4 mm. The time series of each voxel was scaled to have a mean of 100 so that all signal changes are automatically in units of percent difference from the mean.

A voxel-wise generalized linear model (GLM) was used to analyze the MR time series. The GLM included the following regressors of no interest: a third order polynomial (to model baseline fluctuations) and six mean-subtracted motion estimates from the co-registration routine (roll, pitch, yaw; x-, y-, z- translations).

For the main fMRI experiment, two GLMs were constructed using the AFNI program *3dDeconvolve*. The first GLM contained five regressors-of-interest, one for each different physical stimulus type: A, An, AV, AnV, V. The second GLM added regressors based on intelligibility, with only the noisy sentences *post-hoc* sorted by behavioral response into sentences that were rated as intelligible (Y); not intelligible (N); or no response recorded (no resp). This resulted in a total of eight regressors-of-interest (A, An-Y, An-N, AV, AnV-Y, AnV-N, V, no resp).

For the localizer fMRI experiment, one GLM was constructed with two regressors of interest, one for all stimulus blocks containing mouth movements and one for all stimulus blocks containing eye movements.

The regressors of interest were created by convolving the onset time and the duration of each stimulus (3 s trial duration for the main experiment and 30 s block duration for the localizer experiment).

For the main experiment, the time course of the BOLD response for each stimulus type was estimated in a window from stimulus onset to 15 s after stimulus onset using tent (stick) functions. Because the TR was 1.5 s, the resulting impulse response functions (IRFs) contained 11 time points (the first time point at *t* = 0 s post-stimulus was forced to zero).

### 2.8. ROI construction

The cortical surface parcellation provided by FreeSurfer was used as the basis for ROI construction (Fischl et al., 2004). First, the superior temporal gyrus, superior temporal sulcus, and middle temporal gyrus labels (Destrieux et al., 2010) were grouped into a single ROI. These labels classify the entire length of the STG and STS as a single ROI, but speech processing in the anterior and posterior temporal portions are functionally distinct (Ozker et al., 2017, 2018). Therefore, the ROI was divided into anterior and posterior portions using a boundary midway between the most anterior and posterior points of the ROI. Across subjects, the average location of the ROI midpoint was *y* = −25 ± 1.5 mm (left hemisphere) and *y* = −24 ± 0.8 mm (right hemisphere); co-ordinates in MNI standard space (N27). The anatomical pSTG/S ROI was refined with a functional criterion. Only voxels with a significant response to any stimulus, defined as an overall omnibus *F*-test with *F >* 5, *q <* 0.0001, false discovery rate (FDR) corrected; and a significant preference for mouth movements compared with eye movements in the localizer fMRI experiment (*q <* 0.05; FDR corrected) (Zhu and Beauchamp, 2017).

### 2.9. Univariate analysis, multivariate analysis and mixed-effects modeling

The beta coefficient from the GLM (in % BOLD signal change from fixation baseline) for each type of sentence was used as the measure of response amplitude in each voxel. For the univariate analysis, the beta coefficient was averaged across all voxels in each ROI. For multivariate analysis, separate calculations were performed for each ROI. Within each voxel, the response was mean-centered by subtracting the mean response across conditions from the response to each condition (e.g. Haxby et al. 2001). Conditions were compared pairwise using the linear (Pearson’s) correlation of the patterns evoked by each condition. For statistical tests, the correlations were Fisher z-transformed to ensure normality.

Data across participants was analyzed using linear mixed-effects (LME) models created with the *lme4* package in R (Bates et al., 2015) with additional statistical values provided by the *car* and *lmerTest* packages (Kuznetsova et al., 2017). Single values from each participant or hemisphere (percent intelligible for behavioral data; beta coefficients for univariate analysis; Fisher z-transformed correlation coefficients for multivariate analysis) were used as the dependent measure. Participant was entered as a random factor in all models (random intercept but not random slope). For the neural data, hemisphere was entered as a fixed factor (main effect and interaction). Data from all ROIs (both left and right hemispheres) are plotted together in Figs. 3 and 4 to simplify data presentation. Command lines and complete results for all statistical tests may be found in Supplementary Tables 1–3.

## 3. Results

### 3.1. Perceptual data

Participants were presented with five physically different types of sentences in the MR scanner, rating each sentence as intelligible (some or every word in the sentence understood) or unintelligible (no words understood). Consistent difference in intelligibility across conditions were observed (Fig. 1C). Audiovisual sentences (AV) were the most intelligible (99% of sentences rated intelligible), followed by clear auditory-only sentences (A, 84%), audiovisual sentences with pink noise added to the auditory track (AnV, 80%), auditory-only noisy sentences (An, 53%), and visual-only sentences (V, 7%).

Seeing the face of the talker improved perception of both clear and noisy auditory sentences. We examined sentences with an auditory component (AV, A, AnV, An) using a two-by-two LME with fixed factors of modality (audiovisual or auditory) and auditory noise (absent or present) and participant as a random factor. The model formula was *percent_intelligible* ~ *noise* * *modality* + *(1 | participant)*. The model showed significant main effects for modality (*χ*^2^_(1)_ = 32, *p* = 10^−7^) and for noise (*χ*^2^_(1)_ = 45, *p* = 10^−10^) without a significant interaction (*χ*^2^_(1)_ = 2, *p* = 0.1); complete output in Supplementary Table 1.

Sentences were *post hoc* sorted into those rated as intelligible and those rated as unintelligible. Visual-only sentences (V) were almost always rated as unintelligible and clear sentences (AV, A) were almost always rated as intelligible. For noisy sentences (AnV, An) there was a more balanced distribution. Across participants, there was an average of 26 noisy audiovisual sentences rated as intelligible (AnV-Y) and 6 rated as unintelligible (An-N). For noisy auditory sentences, an average of 17 sentences were intelligible (An-Y) and 15 were not (An-N).

### 3.2. Functional localizer: identification of mouth-preferring cortex in pSTG/S

**Fig. 2.**
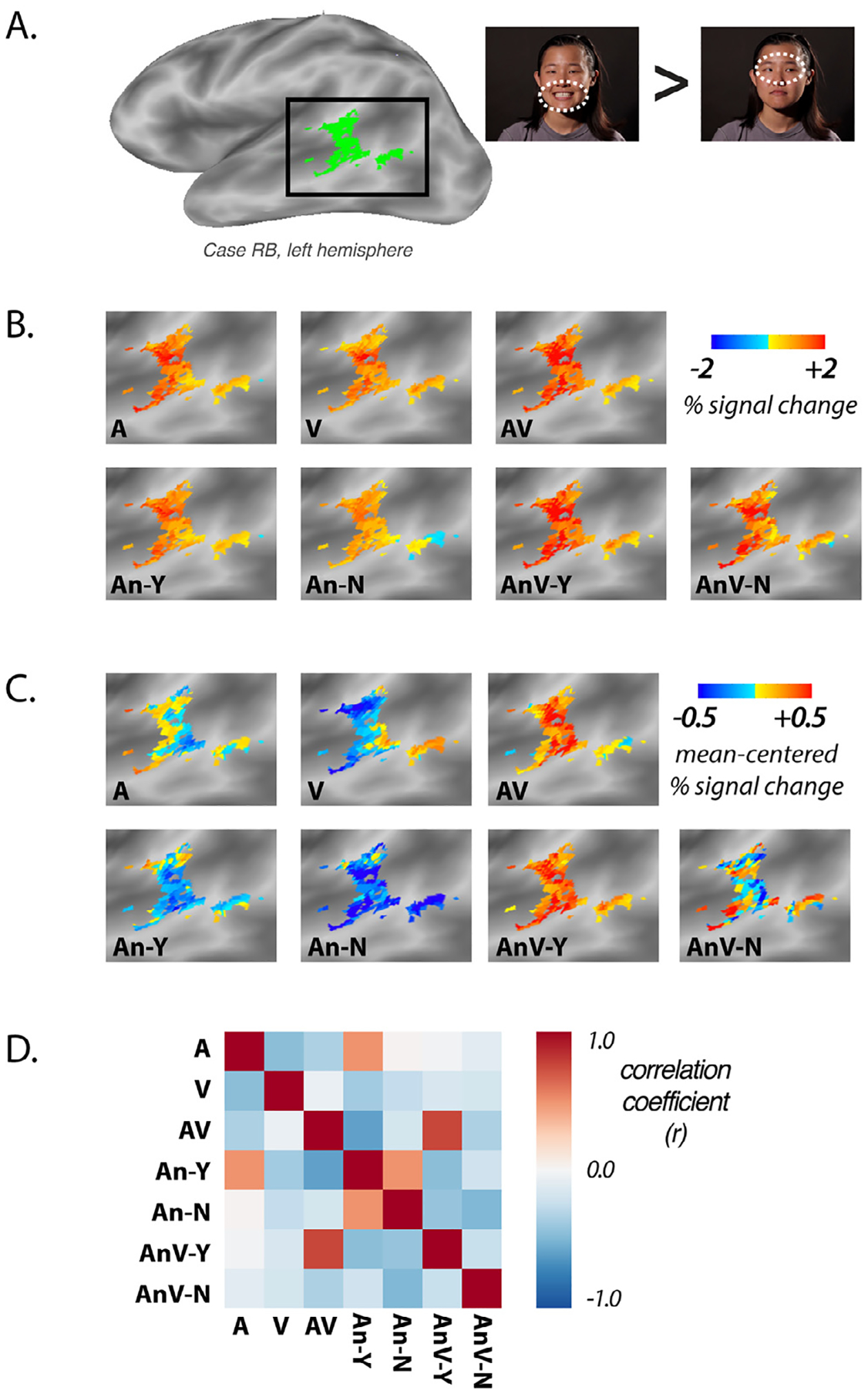
**A**. An independent, block-design fMRI localizer was conducted to create an pSTG/S ROI in the left and right hemisphere of each participant. Silent videos of facial mouth movements and facial eye movements were presented (still frames from each type of video shown, dashed white line highlights moving region of face). Regions in the pSTG/S responding more strongly to mouth-movements videos were selected for the ROI. The ROI for a single hemisphere (case RB, left hemisphere) is shown in green. Black square highlights area shown in ***(B)*** and ***(C)*. B.** In the main experiment, a rapid event-related design with *post hoc* sorting was used to measure the multivariate pattern of responses to different types of sentences. Within the localizer-defined ROI, the response to each of the seven types of sentences was measured. **C.** To accentuate differences between sentence types, the mean response in each voxel across all sentence types was subtracted from the response to each sentence type, producing a mean-centered activation map. **D**. The correlation matrix (Pearson’s *r* coefficient) for all pairs of mean-centered activation maps.

In 43 of 44 hemispheres, the localizer fMRI experiment identified regions in the posterior temporal cortex that responded more strongly to videos of silent mouth movements than to videos of silent eye movements (Fig. 2A). Across participants, the center-of-mass of the pSTG/S ROI in the left hemisphere was (x,y,z) = (−56 ± 0.7, −44 ± 1.3, 10 ± 0.7) (mean ± standard error of the mean across participants) and (54 ± 0.6, −42 ± 1.1, 8 ± 0.6) in the right hemisphere. The mean volume of the ROI was 2575 ± 453 mm^3^ in the left hemisphere and 3178 ± 491 mm^3^ in the right hemisphere.

### 3.3. Multivariate analysis on mean-centered responses

For each hemisphere, an activation map was created showing the pattern of activity evoked in the pSTS/G ROI by each sentence type. As expected, the response to every sentence type was predominantly positive across voxels in the ROI (Fig. 2B). The mean response in each voxel was subtracted from the response to each sentence type, accentuating differences in the response patterns (Fig. 2C). The similarity of the response patterns was quantified by calculating the Pearson correlation between each pair of sentence types using the mean-centered percent change across all voxels in the ROI, producing 21 (_7_C_2)_ pairwise correlations for each hemisphere (Fig. 2D).

**Fig. 3.**
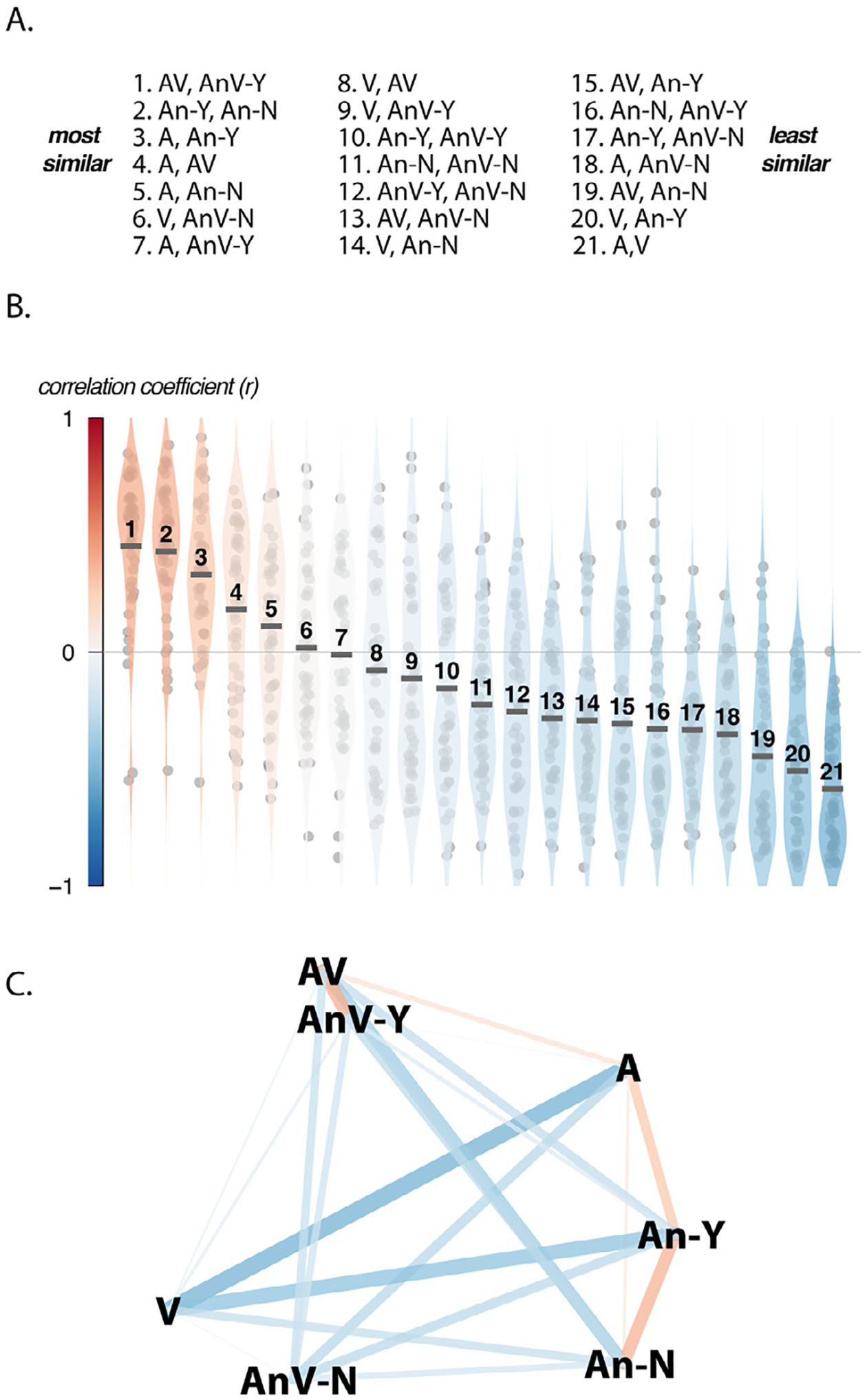
**A**. All pairs of sentence types, ordered from the pair that evoked the most similar response patterns in pSTG/S to the pair that evoked the least similar response patterns. **B.** The correlation coefficients between every pair of sentence types in every hemisphere. Each violin plot represents one pair of sentence types, ordered as in ***(A)***. Circles represent values for each individual hemisphere, black bar represents mean across 43 hemispheres. The outline of the violin shows probability density, the color of the violin corresponds to the mean value (color bar along y-axis.) **C.** Multidimensional scaling (MDS) of the average correlation matrix for all sentence pairs. The location of each sentence type in MDS space is labelled with the name of the sentence type. Lines between sentence types represent the pairwise correlation between that pair of sentences. The color of each line represents the value and sign of the pairwise correlation, same color scale as in ***(B)***. The line width corresponds to the absolute value of the amplitude of the correlation.

There was a wide range of pairwise correlations in the neural response patterns across sentence types and hemispheres, ranging from −0.95 to +0.92. To understand this variability, the pairwise correlation for each sentence type was averaged across the 43 hemispheres in the dataset. The mean correlations were ranked to order the sentence pairs from the pair with the most similar neural responses to the pair with the most dissimilar (Fig. 3A and B).

The sensory modality of the sentence types explained some of the observed variation in pairwise correlations. The most dissimilar neural response patterns (ranked 21st out of 21 pairwise correlations) were evoked by the two sentence types with no sensory modalities in common (A and V sentences; rank 21: mean *r* = −0.59 ± 0.039, standard error of the mean). Sentences that shared the auditory or visual modality had higher mean correlations (AV, A; rank 4, *r* = 0.18 ± 0.055; AV, V; rank 8; *r* = −0.08 ± 0.063).

For noisy audiovisual sentences, intelligibility was a major driver of the evoked response pattern. Despite their physical similarity, there was a large pattern difference between intelligible and unintelligible noisy audiovisual sentences (AnV-Y, AnV-N; rank 12, *r* = −0.25 ± 0.058). To better understand these response patterns, they were compared to the patterns evoked by other sentence types. Noisy but intelligible audiovisual sentences evoked a pattern very similar to that of clear audiovisual sentences (AnV-Y, AV; rank 1: *r* = 0.45 ± 0.048) while noisy but unintelligible audiovisual sentences evoked a pattern most like that of visual-only sentences (AnV-N, V; rank 6: *r* = 0.02 ± 0.054).

The structure of the response patterns was visualized using multidimensional scaling (MDS) on the average pair-wise correlations (Fig. 3C). The MDS plot revealed striking differences between the pattern correlations for audiovisual and auditory-only sentences. While noisy intelligible and unintelligible audiovisual sentences were distant from each other (AVn-Y, AnV-N; pairwise rank of 12, *r* = −0.25 ± 0.058), noisy intelligible and unintelligible auditory-only sentences were nearby (An-Y, An-N; rank 2: *r* = 0.43 ± 0.046).

To quantify this difference between auditory-only and audiovisual sentences, the Fisher-transformed correlations for the *post hoc* sorted sentences were Fisher z-transformed and entered into an LME with stimulus modality (noisy auditory *vs*. noisy audiovisual), intelligibility (Y *vs*. N) and hemisphere (L *vs*. R) as factors. The model formula was *Fz* ~ *intelligibility* * *modality* * *hemisphere* + *(1 | participant)*. There were significant main effects of intelligibility (*χ*^2^_(1)_ = 102, *p <* 10^−16^) and stimulus modality (*χ*^2^_(1)_ = 8, *p* = 0.006) but not hemisphere (*χ*^2^_(1)_ = 0.4, *p* = 0.4). Most importantly, there was a significant interaction between intelligibility and modality (*χ*^2^_(1)_ = 28, *p* = 10^−7^), driven by a greater increase in pattern similarity with intelligibility for audiovisual noisy sentences than for auditory-only noisy sentences. None of the other interactions were significant, complete model output in Supplementary Table 2.

### 3.4. Univariate analysis

**Fig. 4.**
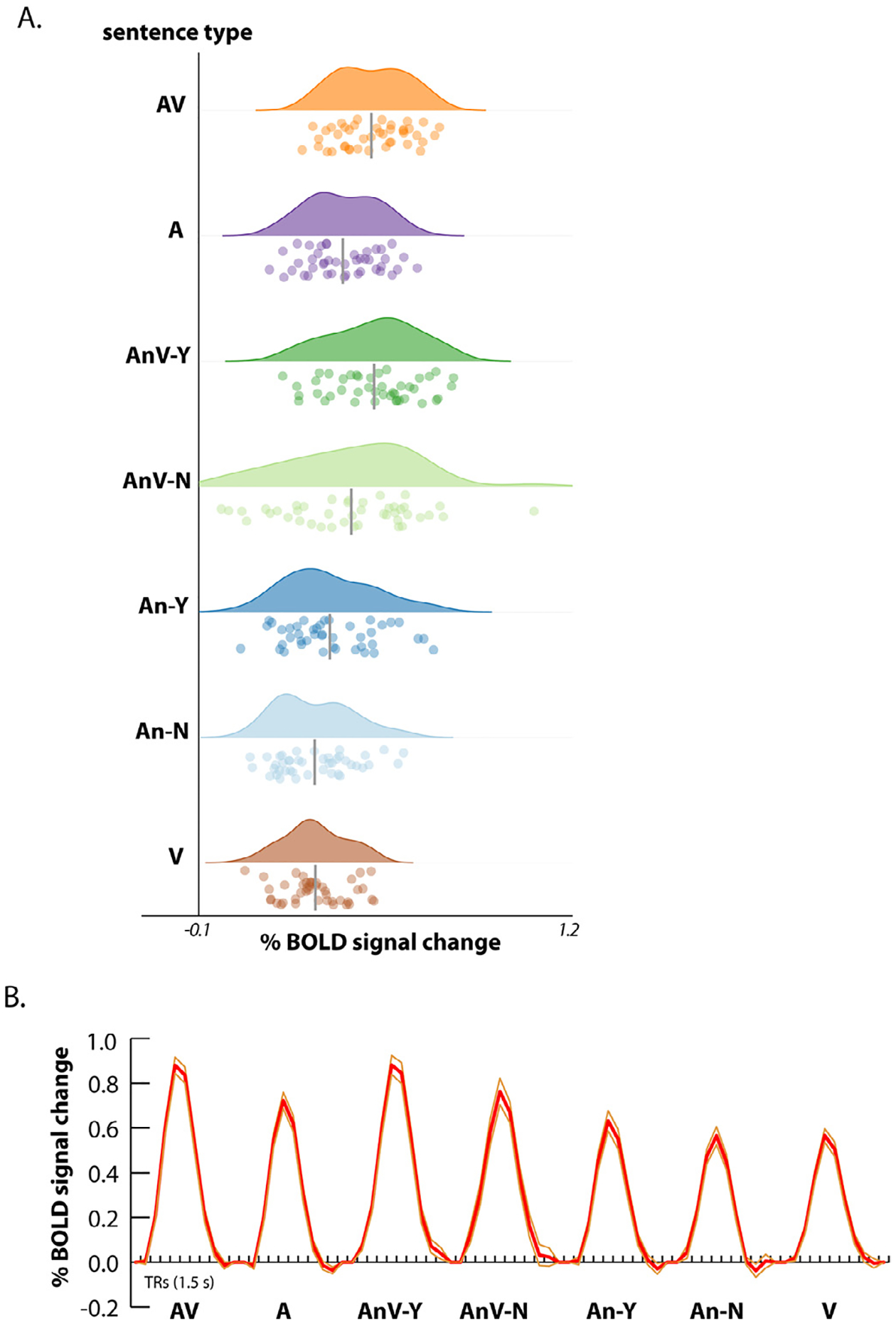
**A**. Raincloud plots showing the distribution of the univariate BOLD response amplitudes across hemispheres for each sentence type (same order as in Fig. 1). For each sentence type, the top plot is the probability density function, the bottom plot shows one symbol per hemisphere, gray line shows mean. **B.** The time course of the BOLD fMRI response to each sentence type (compared with fixation baseline) averaged across voxels in each pSTS/G ROI and then across hemispheres. Thick red lines show the mean across hemispheres, thin orange lines show the standard error of the mean.

We also examined the amplitude of the response in the pSTG/S using a univariate analysis. The mean response to each sentence type was averaged across voxels within each ROI (Fig. 4A).

To quantify the effects of intelligibility, the mean BOLD signal change in each hemisphere was entered into an LME with stimulus modality (noisy auditory *vs*. noisy audiovisual), intelligibility (Y *vs*. N) and hemisphere (L *vs*. R) as factors. The model formula was *betas* ~ *intelligibility* * *modality* * *hemisphere* + *(1 | participant)*. There was a main effect of modality (*χ*^2^ = 50, *p* = 10^−12^) driven by a larger response to audiovisual sentences; a main effect of intelligibility (*χ*^2^ = 11, *p* = 0.0009) driven by a larger response for intelligible than unintelligible sentences; and a main effect of hemisphere (*χ*^2^ = 8, *p* = 0.005) driven by a larger response in the left hemisphere. There were no significant two-way or three-way interactions; complete model output in Supplementary Table 3.

For comparison with previously published studies showing enhanced responses to multisensory *vs*. unisensory speech in pSTG/S, an additional LME was created with sentence type and hemisphere as factors. The model formula was *betas* ~ *sentence_type* * *hemisphere* + *(1 | participant)*. The response to AV sentences was significantly greater than the response to either unisensory auditory sentences (AV *vs*. A, 0.86% *vs*. 0.69%, *t*_266_ = 4.1, *p* = 0.001) or unisensory visual sentences (AV *vs*. V, 0.86% *vs*. 0.52%, *t*_266_ = 8.1, *p <* 10^−16^); values for all pairwise comparisons in Supplementary Table 3.

The impulse response functions (IRFs) for the different sentence types varied in their peak amplitude but showed a similar time course (Fig. 4B). The BOLD signal increased beginning in the first image (collected 1.5 s after stimulus onset) followed by a peak between 4.5 and 6 s after onset, followed by a slow return to baseline.

## 4. Discussion

In individual participants, we used a localizer to identify subregions of the pSTG/S selective for the visual mouth movements that comprise visual speech in individual participants (Rennig and Beauchamp, 2018; Zhu and Beauchamp, 2017). Consistent with decades of behavioral studies, adding visual speech to noisy auditory speech greatly improved intelligibility (reviewed in Peelle and Sommers 2015). *Post hoc* trial sorting was used to measure the pattern of neural responses in the pSTG/S to sentences that were intelligible or unintelligible.

The most surprising result of the present study was that intelligibility was a very strong driver of multivariate response patterns in pSTG/S for audiovisual sentences. Physically similar noisy audiovisual sentences evoked very different BOLD patterns depending on their intelligibility. Noisy audiovisual sentences that were intelligible evoked a response pattern similar to the patterns evoked by audiovisual sentences without any added auditory noise. In contrast, noisy audiovisual sentences that were unintelligible evoked a response pattern most similar to that evoked by visual-only sentences. For auditory-only sentences, the effect of intelligibility was less pronounced: both intelligible and unintelligible noisy auditory sentences evoked similar (but not identical) patterns.

Sensory stimuli that evoke very different neural responses based on their perception as “noise” or “meaningful” has been described in other domains, including visual detection and object recognition (Fisch et al., 2009). This may be due to a “gating” or “ignition” process, in which activity related to meaningful perception spreads widely throughout the brain, while failure to extract meaning results in brain responses that fail to spread beyond early sensory cortex (Beauchamp et al., 2012; Fisch et al., 2009). The similar response patterns evoked by clear and intelligible noisy audiovisual sentences could reflect the successful spread of activity related to meaningful perception, while the similar response patterns evoked by unintelligible audiovisual sentences and visual-only sentences could reflect the failure to perceive something meaningful. Intelligibility modulated the response patterns of audiovisual speech much more than auditory speech, suggesting that the pSTG/S is a key player in the process of using information from the face of the talker to adaptively filter noisy auditory speech.

We also observed smaller pattern differences between intelligible and unintelligible auditory-only speech. This finding is consistent with a previous study that applied multivoxel pattern analysis to auditory-only speech (Okada et al., 2010). Okada and colleagues found that the accuracy of a pattern classifier trained on responses in pSTG/S was high for stimulus manipulations that affected intelligibility (such as spectral rotation) and that classification accuracy in pSTG/S was low for stimulus manipulations that preserved intelligibility while changing acoustic features (such as noise vocoding).

In our study, each sentence was only presented once to each subject, so these results cannot be explained by simple exposure, as in studies of sine wave speech in which the altered speech is intelligible once the clear version has been presented (Benson et al., 2006; Liebenthal et al., 2001).

### 4.1. Multivariate analyses

While many task-based fMRI studies use univariate analysis in which the MR response in each brain location is considered independently, this approach can be criticized as non-biological: the brain uses the distributed pattern of activity in many different neurons to make complex perceptual judgments like those required during speech perception. Instead, multivariate analyses consider the joint activity in populations of voxels (Norman et al., 2006). Pattern classification of auditory cortex fMRI data can successfully distinguish speech features and talkers (De Martino et al., 2008; Formisano et al., 2008); manipulations that influence acoustic features and speech intelligibility (Okada et al., 2010); and different directions of motion for auditory stimuli (Battal et al., 2019) and auditory/visual stimuli (Rezk et al., 2020). A common analysis step in multivariate studies is to mean-center the data in each voxel by subtracting the mean response across conditions, accentuating the difference between conditions (Haxby et al., 2001). This methodological consideration is important in the pSTG/S, where many voxels show a positive response to different types of speech stimuli.

### 4.2. Univariate results

In the univariate analysis, we observed a larger BOLD signal change for clear auditory-only speech compared with noisy auditory-only speech in the pSTG/S, consistent with previous reports of stronger BOLD signals for clear speech throughout lateral temporal cortex (Bishop and Miller, 2009; Evans et al., 2016; Giraud et al., 2004; Stevenson and James, 2009).

In the multisensory domain, pSTG/S responded more strongly to audiovisual speech than to either modality presented alone, consistent with previous studies (Beauchamp et al., 2004b; van Atteveldt et al., 2004; Wright et al., 2003).

For the *post hoc* sorted trials, the univariate pSTG/S response to intelligible sentences was significantly larger than the response to unintelligible sentences, consistent with a previous report that univariate responses in pSTG/S are driven both by the physical stimulus and the resulting percept (Tuennerhoff and Noppeney, 2016).

### 4.3. Functional heterogeneity in pSTG/S

Converging evidence from human fMRI studies and monkey single unit recording show that different subregions of pSTG/S respond more strongly to auditory, visual or audiovisual stimulation (Beauchamp et al., 2004a; Dahl et al., 2009). Our data provides additional support for this finding. For instance, as shown in Fig. 2C for a single hemisphere, more anterior subregions of the ROI showed a stronger response to auditory-only sentences while more posterior subregions showed a stronger response to visual-only sentences.

Another axis of heterogeneity in the pSTG/S is sensitivity to auditory noise. Anterior regions show a diminished response when auditory noise is added to speech, while posterior regions do not, with a sharp divide between the two zones (Ozker et al., 2018; Ozker et al., 2017). Different subregions of pSTG/S also respond preferentially to masked speech (Evans et al., 2016) or different types of social input (Deen et al., 2015) such as eye and mouth movements (Rennig and Beauchamp, 2018; Zhu and Beauchamp, 2017).

For univariate analysis, the signal change across all voxels is averaged, ignoring functional heterogeneity. Multivariate analyses are more sensitive because instead of averaging across voxels, they consider differences in responses to different conditions within individual voxels (Cohen et al., 2017; Kriegeskorte et al., 2008; Norman et al., 2006). For instance, if half of the voxels in an ROI responded exclusively to auditory speech and half responded exclusively to visual speech, the univariate measure (mean response across all voxels) to the two types of speech would be identical, but the multivariate response patterns would be very different.

### 4.4. Syllables *vs*. sentences

Our results differ from a previous fMRI study of audiovisual speech perception which also conducted *post hoc* sorting of trials into intelligible and unintelligible classes (Bishop and Miller, 2009) but did not observed differential univariate BOLD fMRI responses to the two types of trials in pSTG/S. One possible explanation is that Bishop et al. used a stimulus set consisting of noisy syllables, for which the processing demands on pSTG/S may be lower than for words or sentences. The present study and that of Tuennerhoff and Noppeney (Tuennerhoff and Noppeney, 2016) both used a stimulus set consisting of sentences and found effects of intelligibility on univariate measures of BOLD responses in pSTG/S.

### 4.5. The bold impulse response function

The impulse response functions (IRFs) for the different sentence types showed a sharp rise beginning 1.5 s after stimulus onset, a peak at 6 s after onset, followed by a slower return to baseline at about 11 s after stimulus onset, often with a late undershoot in which the signal fell below initial levels. The IRFs showed a strong resemblance to those previously reported for the pSTG/S (Beauchamp et al., 2004b; Nath and Beauchamp, 2012; van Atteveldt et al., 2004; Wright et al., 2003).

Different experimental designs and MR acquisition parameters and could influence the observed IRFs. The present experiment used a rapid event-related (RER) experimental design, in which different stimulus conditions were presented in rapid succession (Burock et al., 1998). Because the BOLD response is much slower than the stimulus presentation rate, responses to successive stimuli overlap and deconvolution was used to extract the IRF (Glover, 1999). A randomization scheme was used to prevent systematic error due to temporal dependencies in which one stimulus type follows another (Dale et al., 1999b). A key advantage of RER designs is that they allow for presenting many more trials within a fixed experimental time, critical for obtaining sufficient statistical power in a study with many experimental conditions. However, deconvolution depends on the assumptions of linearity and time invariance in the BOLD response and may lead to mis-estimation of the IRF compared with slow event-related designs in which there are no overlapping responses (Clark, 2012). For instance, the late post-undershoot may be more difficult to estimate in rapid compared with slow event-related designs TR (Watanabe et al., 2013).

The present experiment used a TR of 1.5 s with no jittering, meaning that the IRFs were estimated on a 1.5 s time base. Estimate the IRF with better temporal resolution could be accomplished by Jittering the stimulus relative to the TR (Watanabe et al., 2013) or with faster MR acquisition techniques (Feinberg and Setsompop, 2013). This would allow more accurate estimation of the time-to-peak of different sentence types; for example, noisy sentences might result in slightly longer latency neural responses than clear sentences.

### 4.6. Summary and future directions

One of the fascinating properties of speech perception is that is categorical, with different stimuli perceived as the same speech element even if they are acoustically very different (Liberman et al., 1957; Pisoni and Lazarus, 1974). Visual speech can strongly influence this categorical perception, even moving a stimulus from one category to another, as in the McGurk effect (Magnotti and Beauchamp, 2017; McGurk and MacDonald, 1976). While BOLD fMRI is too slow to measure the details of the neural response to speech, studies using EEG have shown a hierarchy of multisensory integration effects during perception of audiovisual speech, with visual speech enhancing the representation of both spectrotemporal and phonetic features (O’Sullivan et al., 2021). Surprisingly, the mouth movements made by a talker during speech predict with high accuracy the time-frequency dynamics of audible formants, emphasizing the tight linkage between auditory and visual speech perception (Plass et al., 2020). Joint coding of auditory and visual speech features by neurons in the pSTS/G offer one possible neural mechanism for the perceptual benefit of visual speech on auditory speech perception.

## Supplementary Material

1

## Data Availability

Data and code will be made available as soon as the paper is accepted for publication. Behavioral data and extracted percent signal change as well as R code to analyze these data sets will be uploaded to osf.io.
